# Biallelic variants in *CSMD1* are implicated in a neurodevelopmental disorder with intellectual disability and variable cortical malformations

**DOI:** 10.1038/s41419-024-06768-6

**Published:** 2024-05-30

**Authors:** Elizabeth A. Werren, Emily R. Peirent, Henna Jantti, Alba Guxholli, Kinshuk Raj Srivastava, Naama Orenstein, Vinodh Narayanan, Wojciech Wiszniewski, Mateusz Dawidziuk, Pawel Gawlinski, Muhammad Umair, Amjad Khan, Shahid Niaz Khan, David Geneviève, Daphné Lehalle, K. L. I. van Gassen, Jacques C. Giltay, Renske Oegema, Richard H. van Jaarsveld, Rafiullah Rafiullah, Gudrun A. Rappold, Rachel Rabin, John G. Pappas, Marsha M. Wheeler, Michael J. Bamshad, Yao-Chang Tsan, Matthew B. Johnson, Catherine E. Keegan, Anshika Srivastava, Stephanie L. Bielas

**Affiliations:** 1 Department of Human Genetics, University of Michigan Medical School, Ann Arbor, MI 48109 USA; 2 Advanced Precision Medicine Laboratory, The Jackson Laboratory for Genomic Medicine, Farmington, CTt 06032 USA; 3 Neuroscience Graduate Program, University of Michigan, Ann Arbor, MI 48109 USA; 4 Stanley Center for Psychiatric Research, Broad Institute of MIT and Harvard, Cambridge, MA 02142 USA; 5 Department of Pediatrics, University of Michigan Medical School, Ann Arbor, MI 48109 USA; 6 Medicinal and Process Chemistry Division, CSIR-Central Drug Research Institute, Lucknow, 226031 India; 7 Schneider Children’s Medical Center of Israel, Petah Tikva, 4920235 Israel; 8 Center for Rare Childhood Disorders, Translational Genomics Research Institute, Phoenix, AZ 85004 USA; 9 Department of Molecular and Medical Genetics, Oregon Health and Science University, Portland, OR 97239 USA; 10 Department of Medical Genetics, Institute of Mother and Child, Warsaw, 01-211 Poland; 11 Medical Genomics Research Department, King Abdullah International Medical Research Center, King Saud Bin Abdulaziz University for Health Sciences, Ministry of National Guard Health Affairs, Riyadh, 11481 Saudi Arabia; 12 Department of Life Sciences, School of Science, University of Management and Technology, Lahore, Punjab 54770 Pakistan; 13 Department of Zoology, University of Lakki Marwat, Lakki Marwat, Khyber Pakhtunkhwa 28420 Pakistan; 14 Department of Zoology, Kohat University of Science and Technology, Kohat, Pakistan; 15 Montpellier University, Inserm Unit U1183, Reference Center for Rare Diseases and Developmental Anomalies, CHU, 34000 Montpellier, France; 16 Sorbonne University, Department of Medical Genetics, Hospital Armand Trousseau, 75012 Paris, France; 17 Department of Genetics, University Medical Centre Utrecht, Utrecht University, Utrecht, 3584 EA The Netherlands; 18 Department of Biotechnology, Faculty of Life Sciences, BUITEMS, Quetta, 87300 Pakistan; 19 Department of Human Molecular Genetics, Institute of Human Genetics, Ruprecht-Karls-University, Heidelberg, 69120 Germany; 20 Department of Pediatrics, NYU Grossman School of Medicine, New York, NY 10016 USA; 21 Department of Genome Sciences, University of Washington, Seattle, WA 98195 USA; 22 Department of Pediatrics, University of Washington, Seattle, WA 98195 USA; 23 Brotman Baty Institute, Washington, 98195 USA; 24 Division of Cardiovascular Medicine, University of Michigan, Ann Arbor, MI 48109 USA; 25 Department of Medical Genetics, Sanjay Gandhi Postgraduate Institute of Medical Sciences, Lucknow, Uttar Pradesh 226014 India

**Keywords:** Developmental neurogenesis, Neuronal development

## Abstract

*CSMD1* (*Cub and Sushi Multiple Domains 1*) is a well-recognized regulator of the complement cascade, an important component of the innate immune response. *CSMD1* is highly expressed in the central nervous system (CNS) where emergent functions of the complement pathway modulate neural development and synaptic activity. While a genetic risk factor for neuropsychiatric disorders, the role of *CSMD1* in neurodevelopmental disorders is unclear. Through international variant sharing, we identified inherited biallelic *CSMD1* variants in eight individuals from six families of diverse ancestry who present with global developmental delay, intellectual disability, microcephaly, and polymicrogyria. We modeled *CSMD1* loss-of-function (LOF) pathogenesis in early-stage forebrain organoids differentiated from *CSMD1* knockout human embryonic stem cells (hESCs). We show that CSMD1 is necessary for neuroepithelial cytoarchitecture and synchronous differentiation. In summary, we identified a critical role for CSMD1 in brain development and biallelic *CSMD1* variants as the molecular basis of a previously undefined neurodevelopmental disorder.

## Introduction

The complement pathway is most often studied as a zymogen cascade of soluble and membrane-bound proteins that facilitate the innate immune response. In brief, activation of the complement cascade causes pathogens and foreign materials to be tagged with effector complement fragments that are recognized by their cognate receptors on leukocytes and endothelial cells, triggering pathogen removal [1, 2]. On a molecular level, complement signaling in response to immune activation can be divided into the enzymatic zymogen cascade and the lytic response [1, 2]. The enzymatic cascades are categorized as three distinct pathways: the classical pathway, the lectin pathway, and the alternative pathway [1, 2]. Activation of the lytic response by all three pathways converge on the osmolytic membrane attack complex (MAC) to promote cell lysis, inflammation, and immune cell stimulation [1, 2].

Growing evidence implicates novel emerging functions of complement components in brain development, without activation of a full zymogen cascade or the lytic response. High expression of complement components has been detected in the developing mammalian central nervous system (CNS) [2, 3]. In addition, several complement components are associated with neurodevelopmental disorders (NDDs) [4, 5], neuropsychiatric disorders [6], and neurodegenerative diseases [7, 8]. While classic and lectin pathways have been shown to contribute to the neuropathology of neuropsychiatric disorders and neurodegeneration diseases, similar biology has not been demonstrated during cortical development [1, 2, 9].

Recent work implicates the alternative pathway of complement function in early corticogenesis. C5a complement effector fragment and its cognate receptor C5aR1 are expressed in the neuroepithelium where they function in neuroepithelial polarity and progenitor proliferation [10, 11]. C5aR1 expression is restricted to the apical patch of fate determinants in radial glial neural progenitor cells (NPCs) that line the central lumen of neural rosettes in vitro and the ventricle of the developing cortex in vivo [10, 11]. Activation of the C5a-C5aR1 signaling axis, by administering exogenous C5a, disrupts neuroepithelium polarity and increases NPC proliferation [10, 11]. Inhibition of C5aR1 signaling results in reduced NPC proliferation and neural tube defects in mice [10, 11]. The C3a-C3aR complement effector fragment and cognate receptor pair facilitate radial migration of immature cortical neuronal migration along the basal process of NPCs [12–14]. Knockdown of lectin pathway components C3, MASP1, and MASP2 results in cortical lamination defects with ectopically placed cortical neurons [12]. While overexpression of C3a or an C3aR agonist are able to rescue this phenotype, the partial rescue was also observed with exogenous C3b, a non-lectin pathway fragment that does not bind C3aR nor help form C3 convertase [12]. These findings support a prominent role of the alternative complement pathway in corticogenesis, and suggest its regulation is critical for proper brain development.

CSMD1 is a member of the CSMD family of complement pathway regulators that include CSMD1, CSMD2, and CSMD3. *CSMD* genes are composed of repetitive sequence that encode sequential alternating CUB and Sushi domains, the latter of which are conserved among regulators of the complement pathway. *CSMD1* is highly expressed in brain tissue and associated with neuropsychiatric function. Single nucleotide variants (SNVs) and copy number variants (CNVs) in coding sequence and noncoding regulatory elements of *CSMD1* are risk alleles for attention deficit hyperactivity disorder, schizophrenia, Alzheimer’s disease, and Parkinson’s disease [15–18]. Moreover, emerging *CSMD1* variants have been identified as the molecular basis of autism spectrum disorder (ASD) and a NDD with cerebellar hypoplasia [4, 19–27]. In aggregate, these findings implicate CSMD1 in the brain and in NDDs. CSMD1 can function as a negative regulator of the complement zymogen cascade, but a role for CSMD1 in early human cortical development has not been assessed. However, human cortical neurons generated from biallelic *CSMD1* frameshift (*CSMD1*^*fs/fs*^) hESC lines by directed differentiation show enhanced synaptic deposition of the complement effector fragment C4 [28]. Likewise, in the *Csmd1* knockout (*Csmd1*^*KO*^) mouse, elevated C4 deposition results in synapse engulfment by microglia and excessive pruning. Fewer synapses are correlated to altered cortical circuit formation, without structural brain defects [28]. These findings reveal a role for CSMD1 regulation of complement signaling in synaptic and circuit plasticity.

Here, we present a cohort of individuals with biallelic missense variants in *CSMD1* from seven families with features of global developmental delay (GDD), intellectual disability (ID), dysmorphic facial features, malformations of cortical development (MCD) and seizures. In silico analysis predict these *CSMD1* missense variants function as hypomorphic alleles. To investigate a role for CSMD1 in early human cortical development, as implicated by the clinical features, we generated a forebrain organoid model differentiated from *CSMD1*^*fs/fs*^ hESCs. *CSMD1*^*fs/fs*^ forebrain organoids display disorganized neuroepithelial cytoarchitecture and asynchronous differentiation. The lack of cortical structural defects in *Csmd1*^*KO*^ mice implicates species-specific functional divergence for CSMD1 in the developing mammalian cortex, a finding supported by the intolerance to human *CSMD1* loss of function [28]. This study provides strong evidence for *CSMD1* as a genetic basis of a previously undefined NDD.

## Materials/Subjects and Methods

### Human participants

All individuals, including parents or guardians were consented and enrolled in our institutional review board (IRB)-approved research studies. Consenting was performed in accordance with the ethical standards of the IRB committees on human research participants at the respective institutions [University of Michigan Medical School (MI, USA), Montpellier University Reference Center for Rare Diseases and Developmental Anomalies (France), Translational Genomics Research Institute (AZ, USA), Institute of Mother and Child (Poland), University Medical Centre Utrecht (The Netherlands), NYU Grossman School of Medicine (NY, USA)], and in keeping with international standards. We received and archived written patient consent for all individual patient data included in this manuscript. Permission was obtained for the publication of photos/images from all individuals, or their parents or guardians, whose photos/images are included in this manuscript. Families 2-7 were identified through Matchmaker Exchange, including GeneMatcher and MyGene2 [29, 30].

### Exome-based sequencing

Genomic DNA was extracted from whole blood samples for all participants. Exome libraries from genomic DNA were prepared and captured with the Agilent SureSelectXT Human All Exon 50 Mb Kit. Exome libraries were sequenced on an Illumina instrument at the University of Washington Center for Mendelian Genomics [31] for family 1, Montpellier University Reference Center for Rare Diseases and Developmental Anomalies for family 2, Translational Genomics Research Institute for family 3, the Institute of Mother and Child for family 4, the University Medical Centre Utrecht for families 5-6, and GeneDx for family 7.

### Variant calling and annotation

**Fig. 1:**
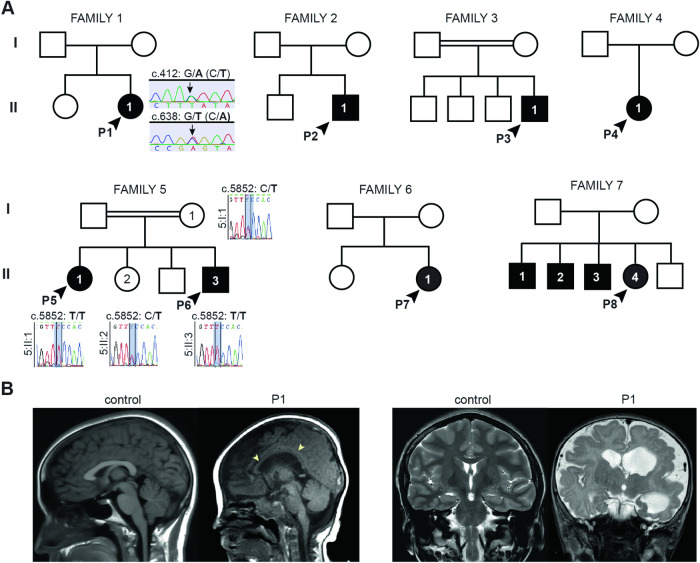
Families with inherited *CSMD1* variants. **A** Pedigree drawings of segregating NDD phenotypes in families 1-7, with generations listed on the left-hand side. Females are represented as circles and males are denoted by squares. Affected family members are indicated by solid black coloring while unaffected are unfilled. Consanguineous partnerships are represented by double lines. Sanger sequencing confirmation was performed on individuals in families 1 and 5 (chromatograms shown). **B** Sagittal (left) and T2 coronal (right) MRIs of P1 at 12 months old (right) relative to control (left). Arrowheads point to thin corpus callosum. Also note normal cerebellar vermis, widened lateral ventricles, and abnormal cortex, suggestive of polymicrogyria.

Reads were aligned to the hg38 reference genome (GRCh38.p13) using Burrows-Wheeler Aligner (BWA). Variant calling of SNVs and CNVs was performed using GATK. The data were filtered and annotated from the canonical *CSMD1* transcript (ENST00000635120.2) using in-house bioinformatics software. Variants were also filtered against public databases, including the 1000 Genomes Project phase 311, Genome Aggregate Database (gnomAD), National Heart, Lung, and Blood Institute Exome Sequencing Project Exome Variant Server (ESP6500SI-V2). Low-quality variants and those with a minor allele frequency >3% were filtered out. Variants in genes known to be associated with MCD were selected and prioritized based on predicted pathogenicity. Reported variants were confirmed by Sanger sequencing of *CSMD1* (NM_033225.6) for P1, P5-P6, and respective family members who submitted samples (Fig. 1A). Pathogenicity of variants was assessed according to American College of Medical Genetics guidelines and using the Franklin Genoox online classification tool (franklin.genoox.com). Splice site predictions were determined using the varSEAK online tool (varseak.bio).

### Human ESC culture

Human ESCs were cultured using feeder-free conditions on Matrigel (Corning with mTeSR-1 (STEMCELL Technologies). H1 (*CSMD1*^*+/+*^, 46XY, WA01, WiCell) and H1 (*CSMD1*^*fs/fs*^, 46XY, WA01, WiCell) ESC lines used in this manuscript were obtained from, and validated by, the Stevens lab at the Broad Institute of MIT and Harvard where cell line quality was assessed by CNV, karyotype, and morphology analyses [32]. Genomic DNA was extracted from ES cell pellets according to the Qiagen DNeasy Blood and Tissue Kit (Qiagen, 69504), including an RNase A step and elution with buffer. PCR was performed with 2 µl of input gDNA, GoTaq Master Mix (Promega M7122), Tm=49, 45 cycles. The following genotyping primers were used: Forward 5’-CTGTGTATTCAAACAGTGCTAA-3’; Reverse 5’-AATCACAGATTAAAGATGGCCAGAA-3’.

The resulting PCR product was treated with ExoSAP-IT (Thermofisher 78200. 200.UL) according to manufacturer instructions. Purified PCR product was submitted for Sanger sequencing at Azenta Life Sciences with forward and reverse primers (separately). The compound heterozygous genotype of *CSMD1*^*fs/fs*^ was confirmed using Synthego ICE CRISPR Analysis Tool (synthego.com/products/bioinformatics/crispr-analysis) (Figure S2A).

### Human neuronal differentiation

*CSMD1*^*+/+*^ and *CSMD1*^*fs/fs*^ hESCs [32] were differentiated into cortical glutamatergic neurons using viral doxycycline-inducible overexpression of NGN2, as previously described [33]. Briefly, on induction day 1 (DIV1), doxycycline hyclate (2 μg/mL) was added to N2 supplemented media (Thermo Fisher, 17502048) with patterning factors SB431542 (Tocris, 1614, 10 μM), XAV939 (Stemgent, 04-00046, 2 μM), and LDN-193189 (Stemgent, 04-0074, 100 nM). On DIV2-6, puromycin selection was performed (5 μg/μL) to remove non-transduced cells. On DIV4, neuronal cells were resuspended in Neurobasal media (Gibco, 21103049) supplemented with B27 (Gibco, 17504044, 50X), doxycycline (2 μg/mL), brain-derived neurotrophic factor (BDNF), ciliary neurotrophic factor (CTNF), and glial cell-derived neurotrophic factor (GDNF) (R&D Systems 248-BD/CF, 257-33 NT/CF, and 212-GD/CF at 10 ng/mL each). Neurons were then maintained as monocultures in this media until collection, as well as co-cultured with murine glial cells derived from early postnatal (P1-P3) mouse brains as described previously [34] (mouse strain https://www.jax.org/strain/100012; animal ethical committee approval by Harvard University: Animal Experimentation Protocol (AEP) # 93-15).

### Forebrain organoid generation

Forebrain organoids were generated based on a previously published Dual-SMAD protocol with few modifications [35]. Briefly, human iPSCs were passaged into 96-well V-bottom shaped ultra-low attachment cell culture plates (PrimeSurface® 3D culture, MS-9096VZ) at a starting cell density of 600 cells per well in 30 µl of mTesR-1 with 1 nM ROCK inhibitor. Cells were counted using LUNA Fluorescent Cell Counting, using a ratio of 18 µl of re-suspended cells to 2 µl of fluorescent dye (Logos Biosystems). After 36 hours, 150 µl of N-2/SMAD inhibition media (cocktail of 1X N-2 supplement (Invitrogen 17502048), 2 μM A-83-01 inhibitor (Tocris Bioscience 2939), and 1 mM dorsomorphin (Tocris Bioscience 309350) in DMEM-F12 (Gibco 11330032)) was added for neural induction. On day 7, embryoid bodies (EBs) were transferred to Matrigel-coated plates to enrich for neural rosettes at a density of 20-30 EBs per well of a 6-well plate, and media was changed to neural differentiation media (0.5X N-2 supplement, 0.5X B-27 supplement (Invitrogen 17504044) with 20 pg/μl bFGF and 1 mM dorsomorphin inhibitor in DMEM/F-12). For organoid differentiation, EBs were outlined on day 14 using a pipet tip and uplifted carefully with a cell scraper to minimize organoid fusion and tissue ripping. Media was changed once more to N-2/B-27 with bFGF only and plates with uplifted organoids were placed on a shaker in the incubator set at a rotation speed of 90. Media changes were performed every 48 hours. Organoid differentiations were repeated in triplicate to generate a minimum of *N* = 100 organoids per genotype for analysis. Images of organoids were captured weekly during differentiation using the EVOS Cell Imaging System (Thermofisher). Cross-section area was measured using Fiji (ImageJ) software. Any fused organoids were excluded from further analysis. Cross-section area was plotted as mean ± SEM using GraphPad Prism (v9.3.1). Statistical analysis was performed on growth curves using simple linear regression F test.

### Western blot analysis

NGN2 cells were lysed with RIPA buffer supplemented with a complete protease and phosphatase inhibitor cocktail (Roche, 04693132001) and collected by scraping. Samples underwent shaking for 10 minutes followed by centrifugation at 15 000 x g for 20 minutes in 4 °C. Protein concentration was quantified by bicinchoninic acid (BCA) assay (Thermo Scientific, 23225). A total of 20 ug of protein was mixed with equal parts 6X Laemmli SDS Sample Buffer (Thermo Scientific, AAJ61337-AC) and boiled for 5 minutes at 95 °C. Samples were loaded onto NuPAGE Tris Acetate 3-8% Gel (Thermo Scientific, EA03752BOX). Proteins were then separated by gel electrophoresis for 15 minutes at 60 V followed by 1 hour at 120 V. Separated proteins were transferred to nitrocellulose membranes (BioRad, 1704158). Blots were probed with rabbit anti-CSMD1 (1:1000, Abcam ab166908) and mouse anti-β-actin (Sigma-Aldrich, A3854; 1:10,000), and goat anti-rabbit IgG (H + L) secondary HRP Antibody (Thermo Scientfic, 31460). Blots were developed using SuperSignal West Femto Plus Chemiluminescent Substrate (Thermo Fisher Scientific, 34095) and visualized using a ChemiDoc Imager (BioRad). Western blot analysis on three biological replicates per genotype (Figure S2). The entire western blot experiment was repeated twice, yielding same results.

### Immunohistochemistry

Human cortical organoids were fixed in 4% PFA for 24 hours at 4 °C, cryoprotected in 15 and 30% sucrose in 1x DPBS for 24 hours at 4 °C, then embedded in OCT with quick freezing in -50 °C 2-methylbutane, followed by cryosectioning at 16 µm. Antigen retrieval was performed on sections by incubation in heated 10 mM sodium citrate solution (95-100 °C) for 20 minutes prior to immunostaining. Sections were then incubated for 1 hour with blocking buffer (5% NDS (Jackson ImmunoResearch) 0.1% Triton X-100, 5% BSA) at room temperature, then overnight with primary antibodies diluted in blocking buffer at 4 °C, and for 1-2 hours in secondary dilution at room temperature. Washes were performed in PBS. For nuclear staining, samples were incubated at room temperature for 10 minutes in Hoechst (1:1000 dilution in PBS) prior to final washes. EdU-labeling was performed using the Click-IT EdU kit (Invitrogen C10337), following manufacturer’s instructions. Primary antibodies used: mouse anti-PAX6 (1:250, Abcam, MA-109), rabbit anti-KI67 (1:200, Abcam ab16667), rat anti-PH3 (1:250, Abcam ab10543), mouse anti-N-Cadherin (1:200, BD Biosciences 610920), rabbit anti-ZO-1 (1:200, Invitrogen 61-7300), rabbit anti-TBR1 (1:200, Abcam ab31940), and rat anti-BCL11B: (1:500, Abcam ab18465). AlexaFluor-conjugated secondaries used: donkey anti-mouse 647 (1:400, Invitrogen A31571), donkey anti-rat 555 (1:400, Invitrogen A48270), and donkey anti-rabbit 488 (1:400, Invitrogen, A21206).

Glass covers were mounted onto all slides with Prolong Gold (Molecular Probes S36972) and incubated for 24 hours at room temperature prior to imaging. Imaging was performed with a Nikon A1ss inverted confocal microscope using NIS-Elements Advanced Research software. Image analysis was performed using Fiji (ImageJ) software. All fused organoids were excluded from further analysis. Borders of neural rosette structures were defined by morphology (radial versus unstructured positioning of nuclei) and biomarker boundaries (N-Cadherin, ZO-1, PAX6, and TBR1). Analysis was performed on 1-3 NRs per organoid. All fluorescent data outliers were excluded from analysis. Outliers were identified using the ROUT method in GraphPad Prism (v9.3.1). For day 56 whole organoid cross-section quantification of BCL11B immunofluorescence (Fig. 4F), organoids at 20X magnification were outlined with the ImageJ freehand draw tool and the region was added as an ‘ROI’. For the BCL11B channel, the threshold tool was used to obtain signal value with the maximum set to 500, distinguishing between autofluorescence and real signal. This percentage subtracted from 100% represents BCL11B and was plotted. Statistical significance of image quantifications was tested using a two-tailed unpaired *t*-test or Mann-Whitney U test, and data was plotted as mean ± SEM using GraphPad Prism (v9.3.1). The variance was determined using the F-test. All data followed a normal distribution per condition, and was determined using Anderson-Darling, D’Agnostino & Pearson, Shapiro-Wilk, and Kolmogorov-Smirnov tests.

### Computational and protein structure analyses

**Fig. 2:**
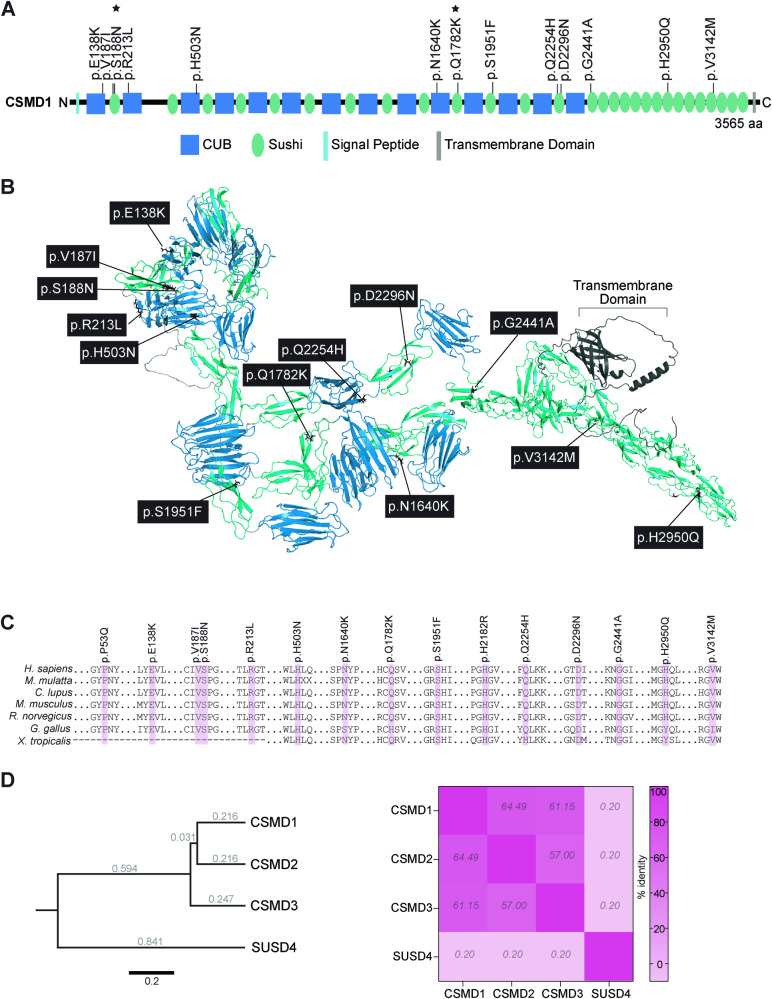
Modeling variant effect on CSMD1 protein. **A** CSMD1 linear protein map with inherited missense variants identified in individuals with NDD. Stars denote inherited *CSMD1* variants not included in our study cohort but previously published. All variants identified by exome and genome sequencing in affected individuals localize to the CUB (blue) and sushi (green) domains of CSMD1. **B** Model of CSMD1 protein structure with mapped clinical variants. Each Cub and Sushi domain was individually modeled using AlphaFold2, and then assembled using DeepAsembly to build the complete CSMD1 structure. Protein domains and variant residues were illustrated using color and licorice functions in PyMOL v2.5.2. **C** Species conservation of affected residues. **D** Rooted phylogenetic tree of CSMD family proteins and the complement regulator SUSD4 as an outgroup (left). Branches are labeled with genetic distance as measured by substitutions per site. Scale bar, 0.2 substitutions per site. Heatmap illustrating percent sequence identity of SUSD4 and CSMD family proteins to each other determined from protein alignment (right).

NCBI HomoloGene tool (https://www.ncbi.nlm.nih.gov/homologene) was used to obtain aligned amino acid sequences of CSMD1 across species at affected residues and flanking regions. Phylogenetic tree of CSMD family proteins with the complement regulator SUSD4 as an outgroup was constructed using the Geneious Prime v2022.1.1 Tree Builder tool. Geneious protein alignment was performed on protein sequences of CSMD family members and SUSD4 using global alignment with free end gaps and a Blosum62 cost matrix. In silico prediction of the functional impact of CSMD1 variants was performed using Polymorphism Phenotyping (PolyPhen-2) v2.2.3r406 using the HumDiv model (http://genetics.bwh.harvard.edu/pph2) or using Sorting Intolerant from Tolerant (SIFT) [36, 37]. Variant effect predictions were also performed using deep learning-based modeling tools DDmut (biosig.lab.uq.edu.au/ddmut) and ESM1b (Figure S1A) [38, 39]. The ESM1b protein language model is unsupervised to minimize bias, and the pre-training data includes a vast array of sequences. DDMut was utilized for forecasting alterations in Gibbs Free Energy by utilizing deep learning models of the localized 3D environment, alongside convolutional layers and transformer encoders, to accurately predict the effects of variants on protein stability. Combined Annotation Dependent Depletion (CADD) Phred scores were obtained for each variant using CADD v1.6 against GRCh38 (https://cadd.gs.washington.edu) [40]. PDB files for CSMD1 (Q96PZ7) were downloaded and extracted from the AlphaFold Protein Structure Database’s reference *Homo sapiens* proteome file #UP000005640 (https://www.alphafold.ebi.ac.uk/download) [41]. All twelve AF-Q96PZ7 fragment model predictions were then assembled into a multi-domain protein structure using DeepAssembly (zhanglab-bioinf.com/DeepAssembly/), which leverages deep learning techniques to consider interdomain interactions [42]. The consensus structure was further annotated in PyMOL v2.5.2 (Fig. 2B). Confidence of the complete CSMD1 structure model was assessed using the Local Distance Difference Test (IDDT) across the protein (Figure S1B). Structure-based protein-binding site prediction was performed using deep learning-based modeling with ScanNet (Spatio-Chemical Arrangement of Neighbors Network) (1.bioinfo3d.cs.tau.ac.il/ScanNet/) [43] (Figure S1C).

## Results

### Biallelic *CSMD1* variants identified in individuals with undefined NDD

Research exome sequencing was performed to provide a molecular diagnosis for a female (P1, 1:II:1) who presented with GDD, moderate-to-severe ID, and focal epilepsy at seven years of age (Fig. 1A). P1 was carried to full-term (39 weeks at birth), though intrauterine growth delay was detected during the pregnancy. At birth, P1 had neonatal hypotonia and microcephaly. Other clinical features included gastroesophageal reflux disease (GERD), drooling, G-tube-dependence, tracheostomy-dependence, dysphagia, upper respiratory infections, eustachian tube dysfunction, and repeated ear infections. Arthrogryposis of hands (ulnar deviation of third-fifth digits and reduced creases on palmar surface), bilateral club feet, and flexion contractures of the knees were noted. Ophthalmological anomalies included amblyopia of the left eye, myopia bilateral, and esotropia. P1 had tonic-clonic, myoclonic-tonic, and myoclonic seizures, that were minimally response to oxcarbazepine, levetiracetam, and CBD oil. Brain MRI at 12 months of age indicated diffusely dysplastic cerebral hemispheres, polymicrogyria, and a thin corpus callosum (Fig. 1B). P1 passed away of Sudden Unexpected Death in Epilepsy (SUDEP) during sleep at eight years of age. Biallelic *CSMD1* variants identified by research exome sequencing emerged as the strongest candidates for the genetic etiology of this NDD. Individual P1 was born to unaffected parents that were heterozygous *CSMD1* carriers of the compound heterozygous *CSMD1* (c.412 G > A, p.E138K; c.638 G > T, p.R213L) missense variants recessively inherited by P1. Analysis of incidental findings revealed compound heterozygous variants in *ABCA1* (NM_005502: c.6202 C > A, p.L2068M; c.3055 G > A, p.V1019I) in P1. Biallelic variants in *ABCA1* are the genetic basis of autosomal recessive Tangier disease (MIM: 205400) characterized by reduced levels of plasma high density lipoproteins (HDL) [44, 45]. These variants are not predicted to account for the neurological findings in this individual, supporting the candidacy of *CSMD1* as a genetic basis of this NDD.

Collaborations facilitated by international gene-sharing efforts identified seven additional individuals with biallelic variants in *CSMD1* and NDDs (Fig. 1 and Table 1). *Individual P2 (2:II:1)*, a male last evaluated at three years of age, presented with mild ID and attention-deficit hyperactivity disorder (ADHD). Prenatal polyhydramnios and preterm labor were noted. P2 was born at 37 weeks of gestation to unaffected parents. P2 experienced an episode of hypoglycemia at birth and possible respiratory arrest requiring a 24-hour hospital stay in the neonatal intensive care. Hypotonia, diffuse joint hypermobility, and a spiral fracture of the right leg were noted. Retractile testes, inguinal hernia, nevus flammeus over the glabella, and eczema were observed. Craniofacial and ophthalmological evaluation revealed hypertelorism, strabismus, and non-paralytic estropia. Brain MRI at 12 months of age was notable for mild bilateral periventricular white matter intensities, reported as remote injury. Magnetic resonance angiogram was normal. Trio exome sequencing identified compound heterozygous *CSMD1* (c.7285+2 T > C; c.6886 G > A, p.D2296N) variants in P2 inherited from heterozygous parents. No additional genetic findings were reported.

**Table 1:** Clinical summary of individuals with CSMD1 biallelic variants in the present study.

Individual	Genetics					Clinical features				
	Allele 1 (NM_033225.6)	Allele 2 (NM_033225.6)	Genotype	Sequencing	Other genetic findings	Development (GDD/ID)	Structural brain defects	Seizures	Musculo-skeletal	Facial dysmorphisms
**P1**	c.412 G > A, p.E138K	c.638 G > T, p.R213L	CH	Research trio exome	*ABCA1* (NM_005502): c.6202 C > A, p.L2068M; c.3055 G > A, p.V1019I	Mild-severe ID	MC (2nd centile), PMG, ACC	+	HYP, ARG	+
**P2**	c.7285+2 T > C	c.6886 G > A, p.D2296N	CH	Research trio exome	none reported	Mild ID	-	-	HYP, DJH	+
**P3**	c.559 G > A, p.V187I	c.559 G > A, p.V187I	HO	Research trio exome	none reported	Moderate ID	MC (2nd centile)	-	HYP	+
**P4**	c.9424 G > A, p.V3142M	c.7322 G > C, p.G2441A	CH	Research trio exome	none reported	GDD; mild ID	MC ( < 2nd centile)	+	CR, ABA	+
**P5**	c.5852 C > T, p.S1951F	c.5852 C > T, p.S1951F	HO	Research trio exome	none reported	Mild ID	N/A	+	ARG, PD, PJL	+
**P6**	c.5852 C > T, p.S1951F	c.5852 C > T, p.S1951F	HO	Research trio exome	none reported	GDD; mild ID	N/A	N/A	N/A	N/A
**P7**	c.8850 C > G, p.H2950Q	c.1507 C > A, p.H503N	CH	Clinical trio exome	*MSH2, CDK11A, SLC35E2* deletions	GDD	-	+	-	+
**P8**	c.4920 C > A, p.N1640K	c.6762 G > C, p.Q2254H	CH	Clinical trio exome	none reported	GDD; mild ID	N/A	-	-	+

*CH* compound heterozygous, *HO* homozygous, *ID* intellectual disability, *GDD* global developmental delay, *MC* microcephaly, *PMG* polymicrogyria, *ACC* agenesis of corpus callosum, *HYP* hypotonia, *ARG* arthrogryposis, *DJH* diffuse joint hypermobility, *CR* cogwheel rigidity, *ABA* advanced bone age, *PD* polyarticular deformations, *PJL* progressive joint limitation, *N/A* not available; +, present; -, absent.

*Individual P3 (3:II:1)*, a male born at 37 weeks of gestation to unaffected parents presented with severe ID, microcephaly, and hypotonia. Craniofacial dysmorphisms included mildly protruding ears, deep-set eyes, ptosis, and broad upper incisors. P3 also presented with a bilateral sandal gap malformation, involving medial displacement of the first toe relative to second, and mild hypertrichosis on his back. Trio exome sequencing identified a homozygous missense *CSMD1* variant (c.559 G > A, p.V187I) in P3 inherited from heterozygous parents. No additional genetic variants were reported.

*Individual P4 (4:II:1)*, a sixteen-year-old female, presented with GDD and mild ID. P4 was carried to full-term and born to unaffected parents. P4 presented with muscular stiffness throughout the range of passive movement, tall stature, long limbs, advanced bone age, and tremor. Craniofacial features included broad forehead, scleral show, and eyebrow synophrys. Brain MRI revealed mild prominence of the ventricles and sulci with volume loss. EEG showed the history of encephalopathy and bilateral slow spike-wave epileptiform activity consistent with generalized epilepsy, but possibly left frontal activity with secondary generalization. EEG at sixteen was within the normal range. Trio exome sequencing detected compound heterozygous *CSMD1* missense variants in P4 (c.9424 G > A, p.V3142M; c.7322 G > C, p.G2441A), inherited from heterozygous parents. No additional genetic variants were reported.

*Individuals P5 (5:II:1) and P6 (5:II:3)* were siblings born to unaffected parents. P5 was a twenty-year-old female who presented with mild ID and was the older sister of P6. There were no prenatal manifestations, and P5 was born with normal birthweight. P5 presented with febrile seizures as a two-year-old but was seizure-free at last examination. P5 had arthrogryposis, polyarticular deformations, progressive joint limitation, and atrophic scars. Craniofacial dysmorphisms include micrognathia, jaw limitation, small mouth, down slanting palpebral fissures, wide nasal bridge, anteverted nostrils, and high-arched palate. P5 also tested positive for anti-RO52KD antibodies, which may indicate autoimmune disease. P6 was the younger brother of P5 and was born at 39 weeks of gestation. P6 presented with GDD and ID. Additional clinical information was not available. Both siblings were homozygous for a *CSMD1* missense variant (c.5852 C > T, p.S1951F) as discovered by exome sequencing. Exome sequencing indicated the unaffected mother and an unaffected sibling were both heterozygous carriers for *CSMD1*:c.5852 C > T, p.S1951F (Fig. 1A). No additional genetic variants were reported.

*Individual P7 (6:II:1)* was an eleven-year-old female who was diagnosed with juvenile myoclonic epilepsy and attention-deficit hyperactivity disorder (ADHD). P9 was born after 38 weeks of gestation to unaffected parents. P9 has had febrile and non-febrile seizures beginning at nine months of age, and she developed absence and tonic-clonic seizures. At age four, EEG revealed photosensitivity and mild epileptiform abnormalities in the frontal lobes. Developmentally, she was not showing evidence of progression, and neuropsychological testing showed evidence of cognitive decline with learning and memory difficulties. Brain MRI did not show evidence of structural defects. Behaviorally, she appeared younger than her biological age. Craniofacial dysmorphisms, including hypertelorism, round face, broad mouth, broad palate, and small hands were noted. Joint mobility was normal. Clinical trio exome sequencing identified inherited compound heterozygous *CSMD1* variants in P9: maternally inherited c.1507 C > A, p. H503N and paternally inherited c.8850 C > G, p.H2950Q. Parents were heterozygous carriers for respective variants. Additional genetic findings included homozygous deletion of *CDK11A* and *SLC35E2* as well as heterozygous inherited deletion of *MSH2*, the latter of which led to the diagnosis of Lynch syndrome.

*Individual P8 (7:II:4)* was a two-year-old female who was diagnosed with developmental delay, intellectual disability, abnormal hand movements, and ASD. Pregnancy was complicated by gestational diabetes. P10 was born at 38 weeks of gestation (weight 2.55 kg; length 47 cm) to unaffected parents. P10 was evaluated by early intervention at seven months old due to motor delay. P10 began walking at 20 months with first words at two years old. P10 qualifies for applied behavior analysis, speech therapy, and physical therapy, and currently communicates by gestures. Occipital-frontal head circumference fell in the 4th percentile (45.5 cm; 4%, Z = -1.73). P10 hearing was normal. Craniofacial dysmorphism included everted lateral third of the lower eyelids. P10 had three older siblings and one younger sibling. Older siblings 8:II:1 and 8:II:2 presented with ASD, and 8:II:1 and 8:II:3 had speech delay. Clinical trio exome sequencing identified inherited compound heterozygous *CSMD1* variants in P10: maternally inherited c.6762 G > C, p.Q2254H and paternally inherited c.4920 C > A, p.N1640K; parents were heterozygous carriers for the respective *CSMD1* variant. No additional genetic variants were reported. Fragile X and microarray testing were negative.

In summary, this biallelic *CSMD1* cohort exhibits shared clinical phenotypes with variable expressivity of GDD, moderate-to-severe ID, structural brain defects, musculoskeletal features, and craniofacial anomalies, and microcephaly (Table 1). Other shared clinical manifestations included seizures, hypotonia, and craniofacial dysmorphisms (e.g., retrognathia, micrognathia, and strabismus). Of note, cases evaluated for MRI in this cohort did not have cerebellar hypoplasia, which has been recently described for individuals with biallelic variants in *CSMD1* [4, 27].

### Bioinformatic analysis of *CSMD1* missense variants indicates recessive zygosity of hypomorphic alleles

*CSMD1* is a large gene, comprised of 70 exons encoding a 3,564 amino acid (388 kD) type-I transmembrane protein. The extracellular portion of CSMD1 consists of several alternating CUB and Sushi domains. We performed in silico analysis to evaluate human *CSMD1* and paralogous genes. *CSMD1* is constrained and intolerant to LOF variation (Observed/Expected (O/E) = 0.51, LOEUF = 0.59) according to gnomAD v4 constraint metrics. *CSMD1* does not exhibit constraints on missense variants in the same database, with the number of observed *CSMD1* missense variants far exceeding the expected (O/E = 1.53; Z = -12.45). Due to high sequence identity between *CSMD* family members, the observed *CSMD1* missense metric may be conflated by variants that should be ascribed to its paralogs—*CSMD2*, in particular (Fig. 2D). Given the mounting human genetics evidence implicating biallelic *CSMD1* missense variants in the etiology of developmental disorders, it will be important to re-evaluate *CSMD* constraint metrics using long-read sequencing data to assign *CSMD* variants to their individual paralogues with high fidelity. The intolerance to LOF suggests that *CSMD1* missense variants represent hypomorphic alleles.

We identified eight individuals with features of NDD and biallelic *CSMD1* variants (Figs. 1–2 and Tables 1–2). One splice site and nine missense variants that localize to the extracellular domain of CSMD1 were identified (Fig. 2A, B). *CSMD1* missense variants affect conserved residues (Fig. 2C). According to a catalog of in silico tools, inherited *CSMD1* missense variants in affected individuals are predicted to disrupt CSMD1 function and are annotated as deleterious or damaging (SIFT: 0.00-0.07; PolyPhen-2 HumDiv score: 0.730-1.00; CADD Phred score:17.46-30) (Table 2). The substituted amino acid for each missense variant represents a marked change in polarity, charge, or pH, which is particularly relevant to the conformational integrity required for transmembrane integration. To further assess *CSMD1* variant pathogenicity, protein analysis was performed using deep-learning language model ESM1b, DDMut, and Deep Assembly. According to these analyses, all variants exhibit varying degrees of pathogenicity. The majority of the *CSMD1* missense variants (9/13) are predicted to be pathogenic using the ESM1b model (with a log-likelihood ratio (LLR) cut-off of -7) [39] (Figure S1A). All remaining variants had ESM1b LLR scores less than -3 (E138K = -5.4, V187I = -3.5, S188N = -4.4, D2296N = -4.8), indicating a slightly deleterious effect (Figure S1A). Analysis by DDMut predicted a negative change of Gibbs free energy for the majority of CSMD1 variants (7/13), indicating a destabilizing effect on CSMD1 (Table 2 and Figure S1A) [38]. DeepAssembly was leveraged to construct the large and highly repetitive full-length CSMD1 protein structure with improved accuracy, as evidenced by the high predicted Local Distance Difference Test (pIDDT) score across the model (Fig. 2B and S1B-S1C) [42]. From this model, three variants (p.N1640K, p.H503N, and p.H2950Q) affect residues located at sites with high predicted probability of interaction, suggesting that a subset of *CSMD1* variants may disrupt important protein-protein interactions (Figure S1C).

**Table 2:** Evidence for *CSMD1* variant pathogenicity from in silico tools, deep learning-based protein modeling, and population data. N/A, not applicable.

Variant (NM_033225.6)	VAF^a^	Homozygous individuals^a^	ACMG Classification	Chain	CADD Phred score^b^	SIFT^b,c^	PolyPhen-2 HDIV^b,d^	DDmut ΔΔG Stability (kcal/mol)^e^	Study
c.412 G > A, p.E138K	0	0	VUS	A	24.1	0	0.998	-0.25	present
c.559 G > A, p.V187I	<0.0005	0	VUS	A	17.5	0	0.414	0.03	present
c.563 G > A, p.S188N	<0.001	0	VUS	A	17.5	0	0.224	-0.09	Costanzo et al., 2022^f^
c.638 G > T, p.R213L	0	0	VUS	A	24.4	0	0.990	0.10	present
c.1507 C > A, p.H503N	<0.0001	0	VUS	A	24.4	0	0.992	0.81	present
c.4920 C > A, p.N1640K	0	0	VUS	A	22.6	0.01	0.634	-0.34	present
c.5852 C > T, p.S1951F	0	0	VUS	A	29.8	0	0.999	-0.04	present
c.5344 C > A, p.Q1782K	<0.005	0	VUS	A	19.2	0.05	0.227	0.05	Costanzo et al., 2022^f^
c.6762 G > C, p.Q2254H	0	0	VUS	A	19.7	0.37	0.001	0.03	present
c.6886 G > A, p.D2296N	<0.00005	0	VUS	A	22.8	0.07	0.948	0.14	present
c.7285+2 T > C	0	0	VUS	N/A	30.0	N/A	N/A	NA	present
c.7322 G > C, p.G2441A	0	0	VUS	A	23.5	0	1.000	-0.70	present
c.8850 C > G, p.H2950Q	0	0	VUS	A	22.0	0.07	0.087	-0.53	present
c.9424 G > A, p.V3142M	<0.00005	0	VUS	A	22.5	0.01	0.730	-0.53	present

^a^gnomAD v3.1.2 controls

^b^GRCh38-v1.6

^c^SIFT scores for missense only, ranging from 0 (deleterious) to 1 (tolerated)

^d^PolyPhen-2 scores for missense only, ranging from 0 (benign) to 1 (probably damaging)

^e^ΔΔG = changed in Gibbs free energy; Negative values indicate destabilizing variants

^f^Costanzo F, Zanni G, Fucà E, Di Paola M, Barresi S, Travaglini L, et al. Cerebellar Agenesis and Bilateral Polimicrogyria Associated with Rare Variants of CUB and Sushi Multiple Domains 1 Gene (CSMD1): A Longitudinal Neuropsychological and Neuroradiological Case Study. Int J Environ Res Public Health 2022, 19(3).

The one splice site variant, c.7285+2 T > C, is predicted to disrupt the 5’ splice donor site of intron 48. Use of the predicted alternative cryptic splice site 46 nucleotides downstream generates a termination codon at amino acid 2431 that truncates CSMD1 after the final CUB domain (Class 5 splicing effect, varSEAK). Together, computational findings provide evidence for negative variant effect predictions for recessive *CSMD1* variants identified in affected individuals. The variability in predicted effects across *CSMD1* variants likely reflects the nuances of hypomorphic effects, especially for long proteins with several protein domains.

The distribution of variants relative to functional CUB and Sushi domains throughout CSMD1 suggest that a subset of amino acids convey critical structural or functional roles. Of note, missense variants in CSMD1 CUB domains (p.E138K, p.R213L, p.503 N) cluster towards the N-terminal extracellular portion of the protein, whereas variants in the Sushi domains are distributed more evenly through the extracellular portion of CSMD1 (p.V187I, p.S188N, p.Q1782K, p.S1951F, p.Q2254H, p.D2296N, p.H2950Q). Individuals with biallelic N-terminal *CSMD1* variants exhibit features of MCD at a higher frequency, as observed in individuals P1 and P3. This is further supported by a recently published case report (Table 1) [4]. In this published case, biallelic *CSMD1* (p.S188N; p.Q1782K) variants present with polymicrogyria, similar to polymicrogyria and thin corpus callosum observed in P1 (p.E138K; p.R213L) [4]. In contrast, biallelic C-terminal *CSMD1* variants (c.7285+2 T > C; p.D2296N), as detected in P2, are associated with mild ID and an unremarkable MRI.

The biallelic *CSMD1* missense variants described here are absent or represented at extremely low frequency (<1.0%) in heterozygous state in the general population, and only 19 homozygous missense variants in *CSMD1* are known to be tolerated (based on the gnomADv3.1.2 control population database) (Table 2). This data is consistent with *CSMD1* functioning as a recessive genetic etiology of NDD. The potential clinical significance of the detected missense variants in our cohort underscores recurrent *CSMD1* missense variants that have been identified in sequencing studies of large ASD cohorts [19–26]. Based on the growing number of rare *CSMD1* variants in individuals with ASD, SFARI Gene has recently classified *CSMD1* as a Level 2, Strong ASD risk gene. Together, population and clinical data implicate essential functions of *CSMD1* in cortical development.

### *CSMD1*^*fs/fs*^ cortical organoids exhibit disorganized neural rosette polarity

**Fig. 3:**
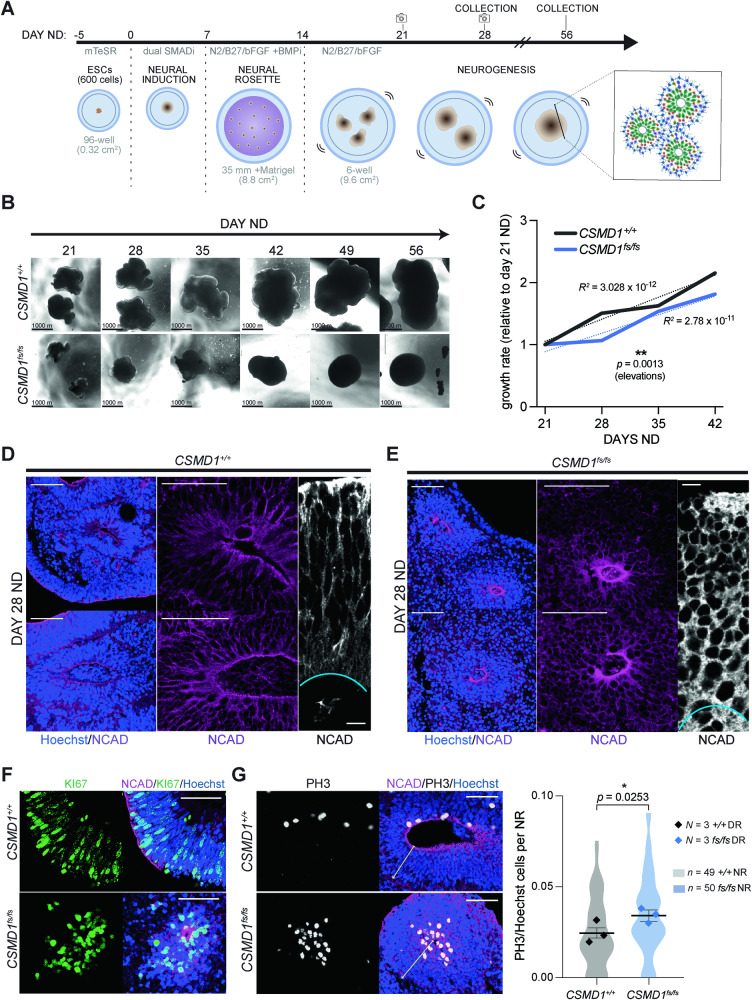
Characterization of neuroepithelium polarity and proliferation in *CSMD1*^*+/+*^ and *CSMD1*^*fs/fs*^ forebrain organoids*.* **A** Schematic of forebrain organoid differentiation, starting with 600 ESCs per well in 96-well V-bottom cell culture plates. Cross-sectional images were captured of live organoid tissue at days 21, 28, 35, and 42 ND. **B** Representative images across developmental time points by genotype of organoids growing in suspension, used for cross-section area growth analysis from day 21 ND to day 42 ND. **C** Growth rate determined by mean relative to day 21 ND, from day 21 ND to day 42 ND (right). *N* = 100-300 organoids from 3 independent differentiations (30–100 organoids per differentiation) per genotype per time point. Statistical significance determined using simple linear regression (elevations, *p* = 0.0013; slopes, *p* =>0.9999). Representative images of NRs across independent organoid replicates to demonstrate gross morphology (Hoescht, N-Cadherin) for *CSMD1*^*+/+*^ (**D**) and *CSMD1*^*fs/fs*^ (**E**). Lower 20X magnification N-cadherin immunostaining showing organization of entire NR (left; scale bar 50 μm); higher magnification of NR (middle; scale bar 50 μm); NR radial segment (right; scale bar 10 μm). KI67 (**F**) and PH3 (**G**) immunostaining of day 28 NRs for each genotype with respective quantifications. Scale bars, 50 μm. Data are shown as points representing differentiation batch means (*N* = 3 per genotype) super-imposed on a violin plot of the distribution of individual NRs (*CSMD1*^*fs/fs*^ *n* = 50; *CSMD1*^*+/+*^ *n* = 49). Error bars represent mean ± SEM for all NRs. Significance determined by unpaired two-tailed *t* test on all NR data (PH3/Hoechst*, p* = 0.0253). Variance, F-test (PH3/Hoechst*, F* = 1.328, *p* = 0.3268).

To investigate the function of CSMD1 in early human cortical development, we generated 3D forebrain organoids. Induced pluripotent stem cell lines reprogrammed from samples derived from individuals biallelic for *CSMD1* missense variants were not available for research. Alternatively, *CSMD1*^*fs/fs*^ hESC lines were created using CRISPR-Cas9 editing that generated compound heterozygous frameshift (fs) variants (19 bp and 20 bp) in exon 2 of *CSMD1* (*CSMD1*^*fs/fs*^) (Figure S2A) [28, 32]. These variants create premature terminations, disrupting CSMD1 expression in *CSMD1*^*fs/fs*^ cortical neurons generated by directed differentiation of hESCs, as indicated by Western blot analysis (Figure S2B) [28]. Thus, analysis of *CSMD1*^*fs/fs*^ organoids allows the loss of CSMD1 during early corticogenesis to be assessed in vitro. We used dual SMAD neural differentiation of *CSMD1*^*+/+*^ control and *CSMD1*^*fs/fs*^ hESCs to generate forebrain organoids that progress through the sequential order and pace of in vivo cortical development (Fig. 3A). Each organoid differentiation was initiated with 600 hESCs per organoid and differentiated for the defined days of neural differentiation (ND). Organoid cross-sectional area was measured weekly over 35 days of ND (excluding any fused organoids) (Figs. 3B and S2C). *CSMD1*^*fs/fs*^ organoids were consistently smaller as compared to *CSMD1*^*+/+*^ control organoids, across three independent neural differentiations, indicating a reduced growth trajectory between genotypes (Figs. 3B, C and S2C).

Neural rosette (NR) structures form within forebrain organoids and recapitulate the polarity and cellular heterogeneity of in vivo neuroepithelium that gives rise to the cerebral cortex. Neuroepithelium is characterized by NPCs that are organized in a pseudostratified configuration, allowing for mitosis of progenitors directly adjacent to the central lumen through interkinetic nuclear migration. These features of NR differentiation were used to assess *CSMD1* neuropathology. NRs generated from three independent organoid differentiations were evaluated. While individual NRs varied in size independent of genotype, *CSMD1*^*fs/fs*^ NRs failed to establish apical-basal polarity characteristic of neuroepithelium (Fig. 3D, E). Immunostaining of adherens junctions (N-Cadherin) highlighted the altered spatial organization of *CSMD1*^*fs/fs*^ NRs. N-Cadherin expression is enriched in the apical patch of NPC cell membranes attached to the central lumen in control *CSMD1*^*+/+*^ NRs. In contrast, *CSMD1*^*fs/fs*^ N-Cadherin was expressed uniformly across the NR tissue (Fig. 3D, E). Likewise, *CSMD1*^*fs/fs*^ NPCs are rounded, without the elongated NPC soma morphology attributed to bipolar apicobasal process attachments that span the width of the neuroepithelium (Fig. 3E).

**Fig. 4:**
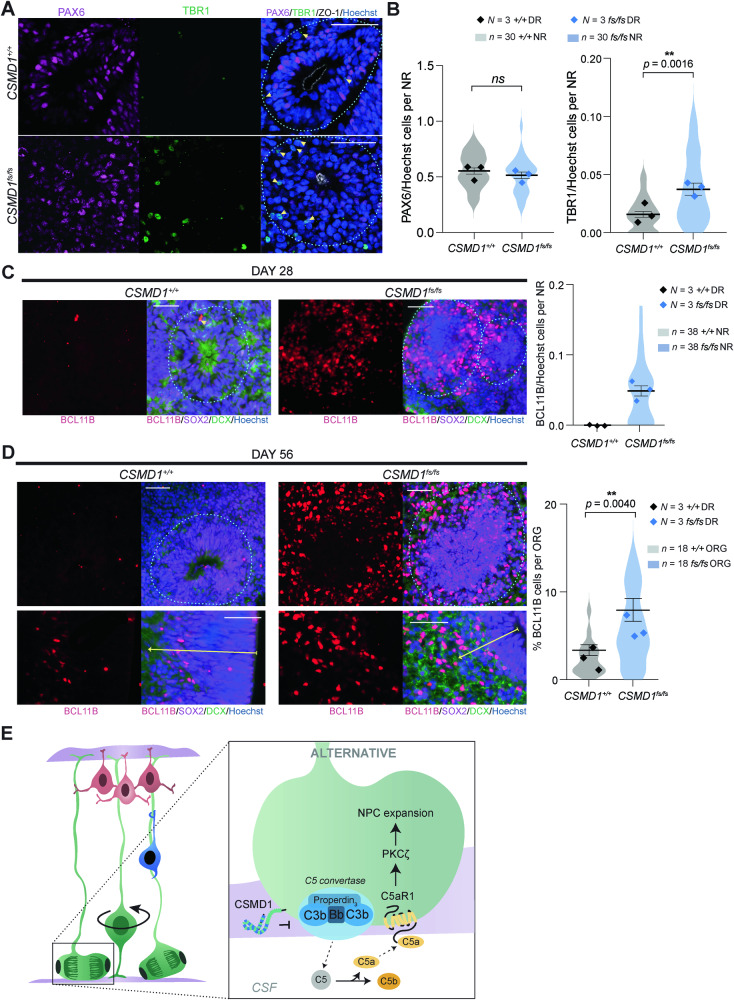
*CSMD1*^fs/fs^ differentiation defects of deep layer cortical neurons in early corticogenesis. **A** Immunostaining of NPCs (PAX6) and early born neurons (TBR1) with quantifications (**B**). Scale bars, 50 μm. Data are shown as points representing differentiation batch means (*N* = 3 per genotype) super-imposed on a violin plot of the distribution of individual NRs (*n* = 30 per genotype). Error bars represent mean ± SEM for all NRs. *ns*, not significant. Significance determined by: unpaired two-tailed *t* test for PAX6/Hoechst (*p* = 0.3524) and Mann-Whitney U test for TBR1/Hoechst (*p* = 0.0016). Variance, F-test (PAX6/Hoechst*, F* = 1.065, *p* = 0.8672; TBR1/Hoechst*, F* = 4.241, *p* = 0.0002). **C** Immunostaining of layer 5 excitatory neurons (BCL11B) in day 28 NR per genotype with quantifications (right). Scale bars, 50 μm. Data are shown as points representing differentiation batch means (*N* = 3 per genotype) super-imposed on a violin plot of the distribution of individual NRs (*n* = 38 per genotype). Error bars represent mean ± SEM for all NRs. Statistical significance was not assessed given the absence of BCL11B cells in control NRs. **D** BCL11B immunostaining in day 56 NRs (left) and percentage of BCL11B cells per whole organoids per genotype (right). Data are shown as points representing differentiation batch means (*N* = 3 per genotype) super-imposed on a violin plot of the distribution of individual organoids (*n* = 18 per genotype). Error bars represent mean ± SEM for all organoids. ORG, organoids. Statistical significance was assessed with Mann-Whitney U test (*p* = 0.0040) given the variance of the data (*F* = 4.498, *p* = 0.0034). **E** Illustration of proposed model of CSMD1 regulation of the alternative complement pathway in NPCs of the developing neuroepithelium via inhibition of C5 convertase functions. *CSF*, cerebrospinal fluid. *Left:* NPCs at the apical surface are green, migrating neurons are blue, and mature neurons at the cortical plate are red.

Consistent with this loss of cytoarchitectural polarity in *CSMD1*^*fs/fs*^ NRs, mitotically active KI67 and PH3 NPCs cluster around the central lumen (Figs. 3F, G and S1D). This distribution is contrasted to the uniform apical and basal distribution of KI67 NPCs across the pseudostratified neuroepithelium of the control *CSMD1*^*+/+*^ NRs (Figs. 3F, G and S1D). Clustering of NPCs around the central lumen is predicted to disrupt interkinetic nuclear migration, which can ultimately alter the balance of symmetric versus asymmetric divisions that directly impact the total amount of NPCs as well as the timing of differentiation [46]. To assess this developmental mechanism, the timing of early born, deep layer cortical neuron differentiation was quantified. The number of multipotent PAX6 NPCs per NR were comparable between *CSMD1*^*+/+*^ and *CSMD1*^*fs/fs*^ NRs (Fig. 4A, B). Meanwhile, a precocious differentiation of two distinct deep layer cortical neuron subtypes (TBR1 and BCL11B) was observed in *CSMD1*^*fs/fs*^ NRs relative to *CSMD1*^*+/+*^ tissue (*p* = 0.0016, Mann-Whitney U test) at day 28 ND (Fig. 4A–C). This substantial difference in deep layer cortical neuron differentiation diminishes by day 56 of ND, suggesting early differentiation phenotypes can be partially compensated for during corticogenesis (Fig. 4D). These findings implicate a critical role for CSMD1 in neuroepithelium polarity and demonstrate a corresponding trend toward asynchronous differentiation during early corticogenesis.

## Discussion

In this work, we report that biallelic variants in *CSMD1* are the genetic basis of a NDD with ID. Ten distinct *CSMD1* variants were identified in 8 individuals from 6 unrelated families, whose exomes did not reveal pathogenic variants in genes previously associated with a described NDD. Individuals in our cohort present with overlapping features of ID and/or GDD, polymicrogyria, microcephaly, epilepsy, and facial dysmorphisms. In silico analysis of *CSMD1* variants supports recessive inheritance of hypomorphic alleles as the genetic mechanism. Analysis of forebrain organoids, differentiated from of *CSMD1*^fs/fs^ hESC lines, revealed defects in neuroepithelium polarity and NPC differentiation, demonstrating an essential role for *CSMD1* in early human corticogenesis consistent with the intolerance of LOF variants in control population databases (gnomAD v3.1.2). Together, these findings provide evidence that biallelic variants in *CSMD1* are good candidates for the genetic basis of NDD.

Our findings add to the growing list of NDDs attributed to CSMD family members. In addition to *CSMD1*, heterozygous de novo and inherited missense *CSMD3* variants were recently described as the genetic basis for a NDD characterized by ID and autism spectrum disorder [5]. Characterization of a *Csmd3* homozygous frameshift mouse model (*Csmd3*^*fs/fs*^) revealed a significant increase of deep cortical layer neurons at the expense of upper layer cortical neurons in *Csmd3*^*fs/fs*^ mice at embryonic (E) day E18 [5]. This phenotype was compounded by defects in dendrite arborization and reduced synapse density, assessed postnatally [5]. *Csmd3*^*fs/fs*^ mice are viable, though they do exhibit growth retardation and abnormal behavioral testing [5]. These findings accentuate the species-specific differential tolerance to CSMD LOF variants, wherein *CSMD1* and *CSMD3* exhibit LOF constraint in humans, yet homozygous frameshift mouse models for both genes are viable. This discrepancy places greater weight on the human population data to support the pathogenicity of *CSMD1* variants. Due to the high sequence similarity between *CSMD1* and *CSMD2* (Fig. 2D), reads from short-read sequencing databases map promiscuously between these paralogs, which may account for the skewed missense constraints calculated for *CSMD1*. Long-read sequencing analysis of human genetic population data will improve missense detection for the CSMD family of genes. Despite this complication, the combination of LOF intolerance, the absence of clinically discovered *CSMD1* variants detected in homozygosity in the general population, and in silico predicted variant effects support the pathogenicity of recessively inherited hypomorphic *CSMD1* alleles. In silico analysis of different amino acid substitutions at affected CSMD1 residues indicates that missense substitutions display a range of predicted effects (Figure S1A), of which not all are detrimental. This signifies that only a subset of *CSMD1* missense variants are pathogenic, and reliable population data will be required to investigate the pathogenicity of monoallelic *CSMD1* variants for NDDs, as has been observed for *CSMD3*.

The role of the complement system in brain function is an emerging field of study. The complement system is necessary for neurogenesis, neuronal migration, synaptic remodeling, and homeostasis in mammalian brain [1, 2]. Spatial transcriptomics of brain tissue from across the lifespan has revealed uneven cell type and temporal expression of complement enzymatic and lytic components [1, 2, 47]. This reveals mechanisms by which complement machinery can be coopted for brain-specific physiological functions. While neurodegenerative disorders are attributed to defects in classic complement inflammation-mediated mechanisms, complement biology regulating brain development is dissimilar to described physiological pathways [1, 2]. Based on transcriptomic profiling, the alternative pathway appears to be the predominant enzymatic cascade during neural development [2, 47]. While not all MAC sub-components are detected by transcriptomics within the same timeframes, evidence supporting that the complement signaling pathway impinges on biology independent of the lytic response [47]. Regardless, it remains unclear how all three complement pathways converge during development, or in the presence of congenital infection. Detailed analysis over time may reveal that maternal infections during pregnancy could compound *CSMD1* developmental defects and account for the broad variable expressivity observed in the *CSMD1* cohort, especially the most severe presentations of the disorder, such as those associated with MCDs.

Evidence that the alternative pathway has an impact on development independent of lytic function is exemplified by C5a–C5aR1 signaling in neural development [2, 10]. In polarized human and mouse neuroepithelium of the developing cortex, C5aR1 expression is enriched in NPCs with subcellular localization at the apical membrane [10]. Binding of C5a activates PKCζ polarity signaling, as opposed to promoting MAC formation [10]. The alternative pathway is distinct from the classical and lectin pathways, in that the pathway exhibits a persistent low level of activity due to a positive feedback loop that is Properdin-dependent [1]. Properdin exhibits high expression in NPCs, highlighting the necessity for negative regulation of this pathway and its target biological processes [1]. CSMD1 is a strong candidate for this inhibitor function as it is a negative regulator of alternative pathway activity by promoting C3b degradation and exhibits high developmental brain expression [48]. Such a function in brain development provides a biological rationale for *CSMD1* as the genetic etiology of a previously undefined NDD.

Overlapping NPC polarity defects between *CSMD1*^*fs/fs*^ organoids and *C5ar1* knockout mammalian phenotypes implicate convergent complement neurobiology. During cortical development, the polarity of NPCs that compose the neuroepithelium informs the symmetry of proliferation and timing of cortical neuron differentiation [49]. Within polarized NPCs that are attached to the ventricular zone in vivo, or the central lumen of neural rosettes in vitro, the allocation of apical fate determinants between the mitotic daughter progeny is an important determinant of mitotic symmetry [46, 50, 51]. During early brain development, C5aR1 exhibits subcellular colocalization with NPC apical fate determinants, and C5a–C5aR1 signaling promotes NPC polarity, proliferation, and multipotency (Fig. 4E) [10]. Transient blockage of C5a and/or C5aR1 in developing mice reduced NPC polarity and proliferation, cell biology phenocopied in the *CSMD1*^*fs/fs*^ NRs [10]. This suggests that negative regulation of the alternative pathway may be required to maintain the polarity necessary for balanced symmetrical-asymmetrical NPC divisions and cortical neuron differentiation (Fig. 4E). This model is consistent with the observed premature differentiation of deep-layer cortical neurons in *CSMD1*^*fs/fs*^ NRs (Fig. 4A–D). This phenotype is starkest at 28 days of ND, and is partially compensated for by day 56 ND, consistent with asynchronous differentiation that can ultimately change the cortical composition of mature cortical neurons subtypes. This also suggests that *Csmd1*^*fs/fs*^ mice may exhibit cortical lamination defects, similar to the *Csmd3*^*fs/fs*^ mouse model.

In summary, our findings implicate CSMD1 in a previously undefined NDD and demonstrate the necessity of CSMD1-dependent regulation of the complement pathway for proper human neural development. Future investigation of later-stage corticogenesis in CSMD1-depleted models in conjunction with molecular investigation of clinical *CSMD1* variants are promising avenues for revealing novel CSMD1 and complement developmental biology.

### Supplementary information

Supplemental Material

Original Data

## Data Availability

All data are available from the corresponding author on request.
