# Supplementary material for: Biallelic variants in CSMD1 are implicated in a neurodevelopmental disorder with intellectual disability and variable cortical malformations
Source: Cell Death Dis. 2024 May 30;15(5):379. doi: 10.1038/s41419-024-06768-6 (PMC11140003; doi:10.1038/s41419-024-06768-6)
Supplement: Supplementary file 1 — Supplemental Material [file 41419_2024_6768_MOESM1_ESM.docx]

**SUPPLEMENTAL MATERIAL**

**for**

**Biallelic variants in *CSMD1* are implicated in a neurodevelopmental disorder with intellectual disability and variable cortical malformations**

Elizabeth A. Werren^1,2^, Emily R. Peirent^3^, Henna Jantti^4^, Alba Guxholli^1,5^, Kinshuk Raj Srivastava^6^, Naama Orenstein^7^, Vinodh Narayanan^8^, Wojciech Wiszniewski^9^, Mateusz Dawidziuk^10^, Pawel Gawlinski^10^, Muhammad Umair^11,12^, Amjad Khan^9,13^, Shahid Niaz Khan^14^, David Geneviève^15^, Daphné Lehalle^16^, K.L.I. van Gassen^17^, Jacques C. Giltay^17^, Renske Oegema^17^, Richard H. van Jaarsveld^17^, Rafiullah Rafiullah^18^, Gudrun A. Rappold^19^, Rachel Rabin^20^, John G. Pappas^20^, Marsha M. Wheeler^21^, Michael J. Bamshad^22,23^, Yao-Chang Tsan^24^, Matthew B. Johnson^4^, Catherine E. Keegan^1,5^, Anshika Srivastava^25*^, Stephanie L. Bielas^1,5*^

^*^**These authors contributed equally to this work**

^1^Department of Human Genetics, University of Michigan Medical School, Ann Arbor, Michigan, 48109, USA

^2^Advanced Precision Medicine Laboratory, The Jackson Laboratory for Genomic Medicine, Farmington, Connecticut, 06032, USA

^3^Neuroscience Graduate Program, University of Michigan, Ann Arbor, Michigan, 48109, USA

^4^Stanley Center for Psychiatric Research, Broad Institute of MIT and Harvard, Cambridge, Massachusetts 02142, USA

^5^Department of Pediatrics, University of Michigan Medical School, Ann Arbor, Michigan, 48109, USA

^6^Medicinal and Process Chemistry Division, CSIR-Central Drug Research Institute, Lucknow, 226031, India

^7^Schneider Children’s Medical Center of Israel, Petah Tikva, 4920235, Israel

^8^Center for Rare Childhood Disorders, Translational Genomics Research Institute, Phoenix, Arizona, 85004, USA

^9^Department of Molecular and Medical Genetics, Oregon Health and Science University, Portland, Oregon, 97239, USA

^10^Department of Medical Genetics, Institute of Mother and Child, Warsaw, 01-211, Poland

^11^Medical Genomics Research Department, King Abdullah International Medical Research Center, King Saud Bin Abdulaziz University for Health Sciences, Ministry of National Guard Health Affairs, Riyadh, 11481, Saudi Arabia

^12^Department of Life Sciences, School of Science, University of Management and Technology, Lahore, Punjab, 54770, Pakistan

^13^Department of Zoology, University of Lakki Marwat, Khyber Pakhtunkhwa, 28420, Pakistan

^14^Department of Zoology, Kohat University of Science and Technology, Kohat, Pakistan

^15^Montpellier University, Inserm Unit U1183, Reference Center for Rare Diseases and Developmental Anomalies, CHU, 34000, Montpellier, France

^16^Sorbonne University, Department of Medical Genetics, Hospital Armand Trousseau, 75012, Paris, France

^17^Department of Genetics, University Medical Centre Utrecht, Utrecht University, Utrecht, 3584 EA,The Netherlands

^18^Department of Biotechnology, Faculty of Life Sciences, BUITEMS, Quetta, 87300, Pakistan

^19^Department of Human Molecular Genetics, Institute of Human Genetics, Ruprecht-Karls-University, Heidelberg, 69120, Germany

^20^Department of Pediatrics, NYU Grossman School of Medicine, New York, New York, 10016, USA

^21^Department of Genome Sciences, University of Washington, Seattle, Washington, 98195, USA

^22^Department of Pediatrics, University of Washington, Seattle, Washington, 98195, USA

^23^Brotman Baty Institute, Seattle, Washington, 98195, USA

^24^Division of Cardiovascular Medicine, University of Michigan, Ann Arbor, MI, 48109, USA

^25^Department of Medical Genetics, Sanjay Gandhi Postgraduate Institute of Medical Sciences, Lucknow, Uttar Pradesh, 226014, India

*Correspondence: Anshika Srivastava ([asrivastavapgi@gmail.com](mailto:asrivastavapgi@gmail.com)); Stephanie L. Bielas (sbielas@umich.edu)

**This file includes the following supplemental material:**

- Supplemental Figures S1-S2


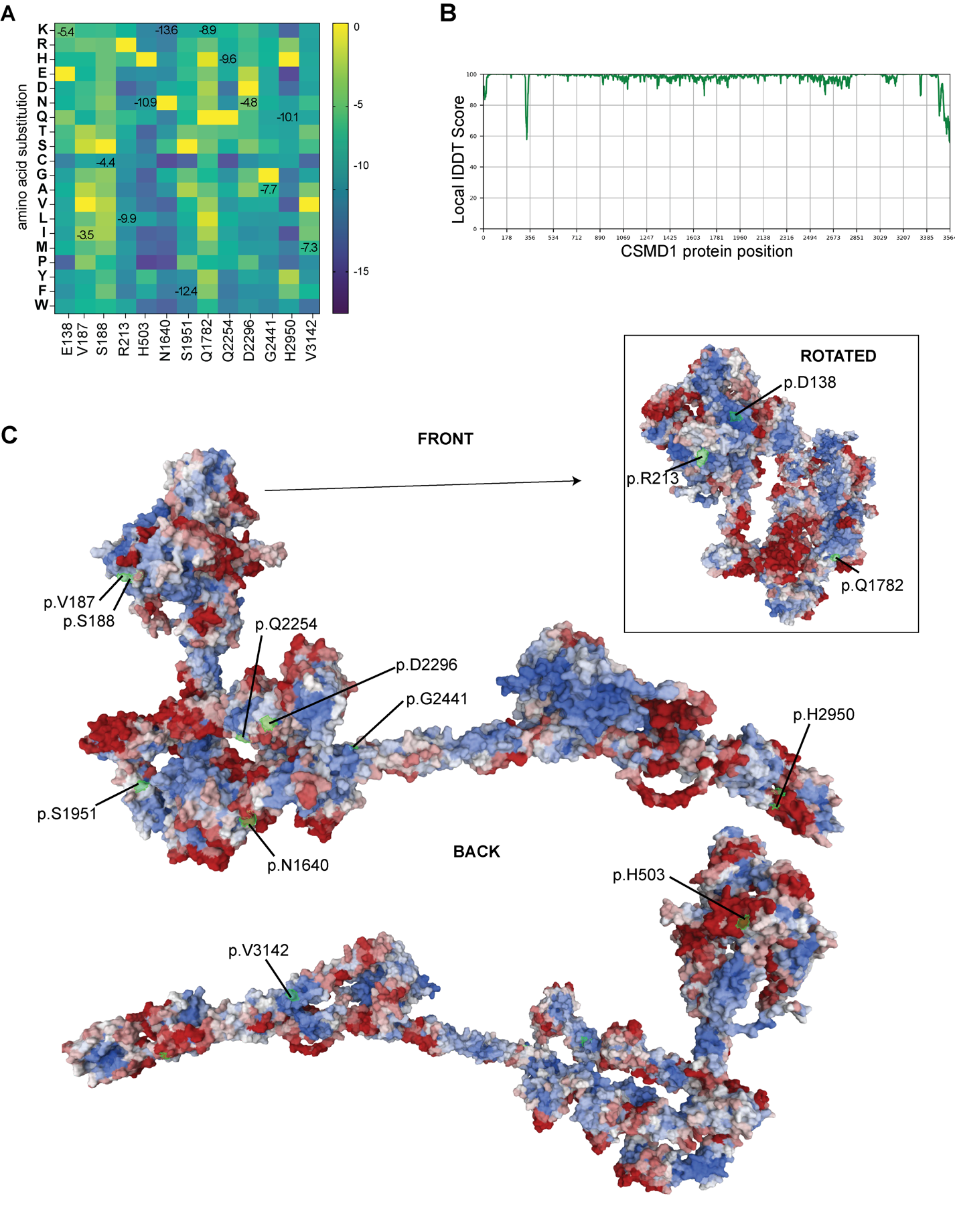


**Figure S1. CSMD1 variant modeling.** (A) Heatmap of variant effect predictions for all possible missense substitutions at CSMD1 affected residues using ESM1b deep learning modeling (scores = log-likelihood ratio (LLR) scores between variant and wildtype residue, where 0 indicates benign effect; LLR < -7 considered pathogenic). (B) Predicted Local Distance Difference Test (pIDDT) score of CSMD1 indicating confidence of structure prediction. (C) Localization of clinical variants (green outline) with respect to deep learning-based predictions of CSMD1 protein-protein interaction interface. Red indicates sites with high probability interaction sites; blue indicates low probability interaction sites.

**
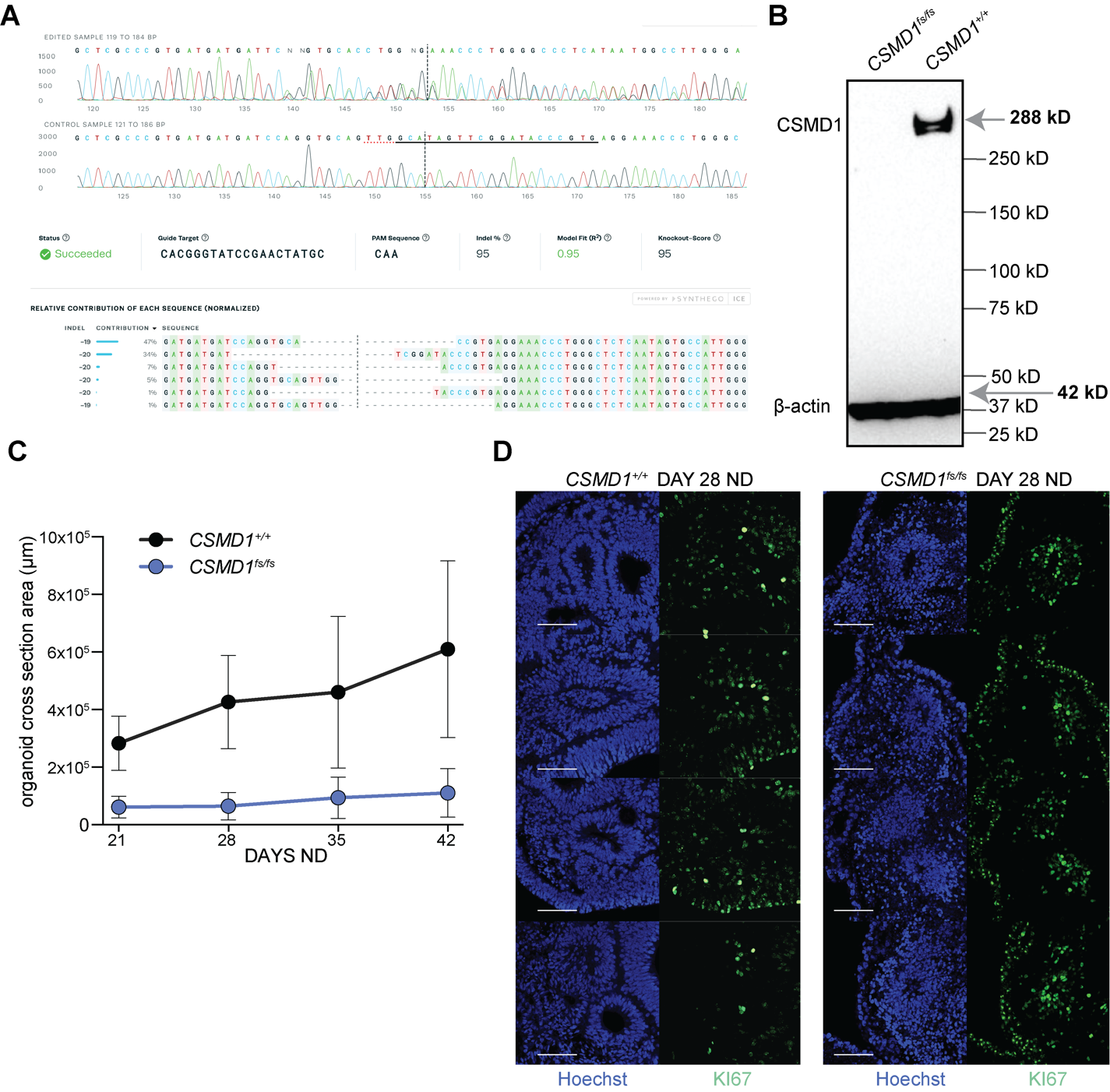
**

**Figure S2.** **Generation of organoid disease models using *CSMD1^fs/fs^* ESC lines.** (A) Sanger sequencing chromatograms and Synthego ICE CRISPR analysis results on Sanger sequencing data from *CSMD1^fs/fs^* and control *CSMD1^+/+^* ESCs confirming compound heterozygous genotype in CRISPR/Cas9-edited cells. (B) Western blot analysis of *CSMD1^+/+^* and *CSMD1^fs/fs^* neurons, incubated with anti-CSMD1 (288 kD) and anti-β-actin as an internal control (42 kD). (C) Organoid growth analysis of cross-section area per genotype (*CSMD1^fs/fs^*, *CSMD1^+/+^*) shown as weekly measurements from day 21 in neural differentiation (ND) to day 42 ND, mean±SD. (D) Representative images of NRs across independent organoid replicates to demonstrate morphology and proliferative differences (KI67) for *CSMD1^+/+^* (left) and *CSMD1^fs/fs^* (right). Scale bars, 50 μm.
