# Supplementary figures and images for: Biallelic variants in CSMD1 are implicated in a neurodevelopmental disorder with intellectual disability and variable cortical malformations
Source: Cell Death Dis. 2024 May 30;15(5):379. doi: 10.1038/s41419-024-06768-6 (PMC11140003; doi:10.1038/s41419-024-06768-6)

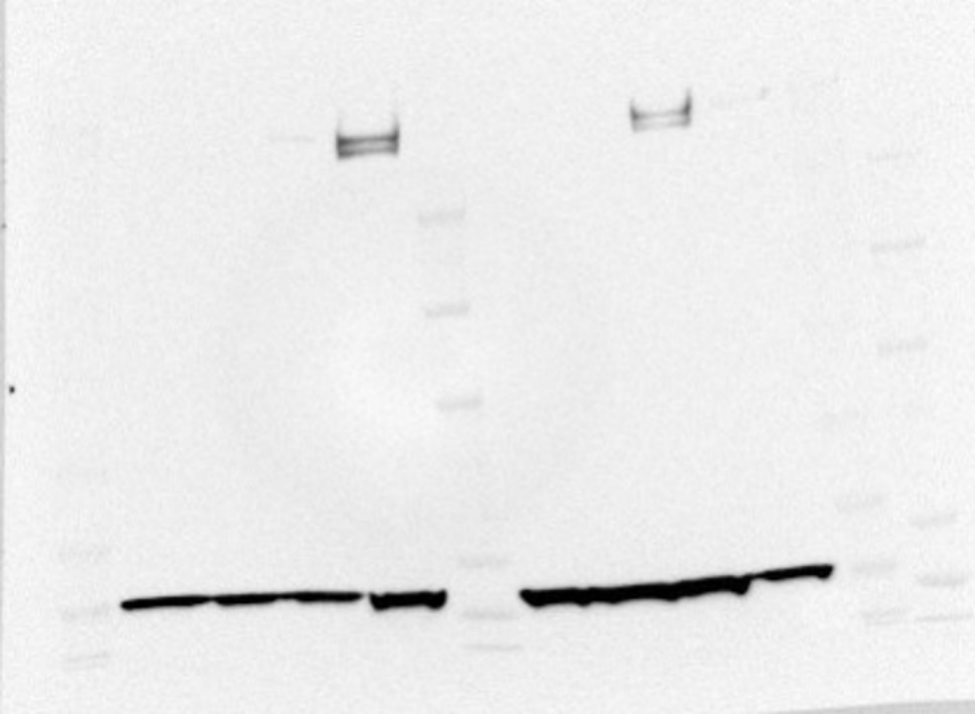

Supplement: Supplementary file 2 — Original Data [file 41419_2024_6768_MOESM2_ESM.pdf]
